# Modern Polycystic Ovary Syndrome (PCOS) Management: Intelligent Drug Delivery and Metabolic Reprogramming for Ovarian Restoration and Fertility Optimization

**DOI:** 10.3390/biom16050626

**Published:** 2026-04-23

**Authors:** Abdel Halim Harrath, Maroua Jalouli, Mohammed Al-Zharani, Md Ataur Rahman

**Affiliations:** 1 Department of Zoology, College of Science, King Saud University, Riyadh 11451, Saudi Arabia; hharrath@ksu.edu.sa; 2 Department of Biology, College of Science, Imam Mohammad Ibn Saud Islamic University (IMSIU), Riyadh 11623, Saudi Arabia; mejalouli@imamu.edu.sa (M.J.); mmylzahrani@imamu.edu.sa (M.A.-Z.); 3 Department of Oncology, Karmanos Cancer Institute, Wayne State University, Detroit, MI 48201, USA

**Keywords:** polycystic ovary syndrome (PCOS), ovarian restoration, intelligent drug delivery, metabolic reprogramming, nanoparticle therapeutics, fertility optimization

## Abstract

Polycystic ovarian syndrome (PCOS) is a complex endocrine and metabolic disorder that affects reproductive health, metabolic function, and long-term cardiovascular health in women of reproductive age. The syndrome is characterized by hyperandrogenism, chronic anovulation, insulin resistance, oxidative stress, and ovarian microenvironment remodeling. While current treatments focus on symptom relief through hormone regulation, insulin sensitizers, or ovulation induction, there is a need to target the underlying molecular and cellular processes that drive disease progression and infertility. Breakthroughs in reproductive and metabolic medicine have led to the development of next-generation therapeutics for PCOS that aim to restore ovarian function at the molecular level. Nanoparticle- and nanofiber-based drug delivery systems offer targeted delivery to the ovaries, improved bioavailability, and controlled release of insulin sensitizers, antioxidants, and anti-androgens. Metabolic reprogramming strategies that target insulin resistance, mitochondrial dysfunction, and autophagy have emerged as potential disease-modifying interventions. In addition, AI-enabled precision medicine approaches are reshaping PCOS management through phenotype-based classification, predictive modeling, and personalized fertility optimization. In this review, we highlight recent advancements in understanding the molecular pathophysiology of PCOS and introduce novel therapeutics that harness intelligent drug delivery, ovarian microenvironment restoration, and AI-based interventions. We discuss the potential of these innovative strategies to update PCOS management options for long-term ovarian restoration and fertility.

## 1. Introduction

Polycystic ovary syndrome (PCOS) is a common endocrine disorder, with a worldwide prevalence of approximately 8–15% in women of reproductive age [1]. Characterized by its reproductive, metabolic, psychological, and cardiovascular consequences, PCOS is a heterogeneous disorder that is clinically and diagnostically complex to manage [2]. While PCOS is characterized by a triad of clinical or biochemical hyperandrogenism, ovulatory dysfunction, and polycystic ovarian morphology, its underlying pathophysiology is far more complex than these diagnostic features.

PCOS is caused by aberrant signaling between the hypothalamic-pituitary-ovarian (HPO) axis, insulin resistance, ovarian microenvironment dysfunction, oxidative stress, inflammation, and impaired autophagy [3]. In the central nervous system, increased GnRH pulsatility preferentially promotes LH secretion over FSH, resulting in theca cell hyperandrogenism and granulosa cell maturation failure, leading to follicular arrest [4]. Insulin resistance, which is also present in the majority of PCOS patients, independent of body mass index (BMI), further contributes to ovarian androgen production and suppression of sex hormone-binding globulin, resulting in higher free androgen levels [5].

In addition to the systemic endocrine abnormalities, there is a growing body of literature surrounding PCOS as a local ovarian disease. Reactive oxygen species (ROS), mitochondrial dysfunction, inflammation, fibrosis, and autophagy dysregulation have all been shown to contribute to the PCOS phenotype [6]. In combination, these abnormalities lead to alterations in oocyte quality, follicular turnover, and endometrial receptivity, which is why ovulation induction [7], while it may result in short-term fertility, does not appear to impact long-term ovarian health.

Although combined oral contraceptives, insulin sensitizers, and ovulation induction agents remain integral to the treatment of PCOS, many of these therapies work primarily to control symptoms, rather than reversing or improving ovarian function [8]. With that, the field has focused on the development of next-generation therapies that can target core pathogenic processes to improve PCOS symptoms. Intelligent drug delivery, metabolic reprogramming, and artificial intelligence (AI)-guided precision medicine are rapidly growing fields that show promise in improving some of the inherent limitations of traditional therapy [9]. This review discusses these emerging approaches and their potential role in future PCOS management, aiming to transition therapy from short-term symptom control toward sustained ovarian restoration and optimized fertility outcomes.

## 2. Methodological Approach

A literature search was conducted using primary biomedical databases, including PubMed, Scopus, and Web of Science. We employed a combination of terms such as “polycystic ovary syndrome,” “PCOS,” “insulin resistance,” “hyperandrogenism,” “ovarian microenvironment,” “oxidative stress,” “autophagy,” “nanoparticle drug delivery,” “metabolic reprogramming,” and “artificial intelligence in reproductive medicine” for our search. To ensure the information remained pertinent, we exclusively incorporated pieces from the past 10 to 15 years. Nonetheless, we incorporated fundamental mechanistic investigations which were deemed suitable.

## 3. Molecular Mechanisms and Clinical Signs of PCOS

PCOS is a polygenic disorder that has hormonal, metabolic, genetic, and environmental contributors. The abnormal interaction between the hypothalamic-pituitary-ovarian axis, insulin resistance, oxidative stress, and ovarian microenvironment results in anovulation and infertility.

### 3.1. Endocrine and Hormonal Dysregulation

**Figure 1:**
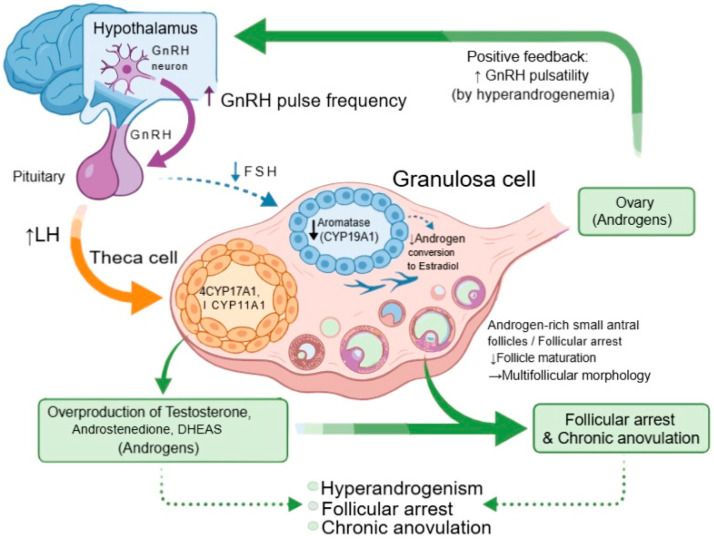
Molecular mechanisms of PCOS in endocrine and hormonal dysregulation. Neuroendocrine-ovarian feedback mechanisms are responsible for hormonal imbalance in polycystic ovary syndrome (PCOS). Elevated hypothalamic GnRH pulse frequency selectively augments luteinizing hormone (LH) secretion while inhibiting follicle-stimulating hormone (FSH) release from the pituitary gland. Increased LH activates ovarian theca cells, enhancing the expression of steroidogenic enzymes (CYP17A1 and CYP11A1) and facilitating the overproduction of androgens, such as testosterone, androstenedione, and DHEAS. Decreased FSH levels hinder granulosa cell aromatase (CYP19A1) function, restricting androgen conversion to estradiol and interrupting follicular development. The buildup of androgen-rich tiny antral follicles results in follicular arrest and multifollicular ovarian morphology. Hyperandrogenemia further stimulates the hypothalamus, enhancing GnRH pulsatility and sustaining endocrine dysregulation. These modifications create a self-perpetuating cycle of hyperandrogenism, disrupted folliculogenesis, and persistent anovulation that defines the pathophysiology of PCOS.

Dysregulation of endocrine and hormonal factors is the underlying molecular pathophysiology of PCOS [10]. The associated reproductive, metabolic, and clinical features are a consequence of the altered feedback interactions between the HPO axis, insulin, and other ovarian local factors, which are thought to be at the center of pathogenesis, causing hyperandrogenism, follicular arrest, and chronic anovulation. It is thought that molecular dysfunction first arises in the hypothalamus, with abnormally high pulsatility of gonadotropin-releasing hormone (GnRH) neurons [11]. These neurons are stimulated at a high frequency, leading to the secretion of GnRH, which selectively promotes the transcription and translation of LH over FSH from the pituitary gland [12]. This results in the observation that many women with PCOS have an increased LH/FSH ratio, one of the endocrine characteristics of the disorder. In the ovary, this means that LH, whose main target cells are theca cells, is the dominant gonadotropin, and exposure to high levels of LH causes a significant upregulation of steroidogenic enzymes such as CYP17A1 and CYP11A1 that are responsible for androgen synthesis [13]. This results in an overproduction of testosterone, androstenedione, and DHEAS by theca cells, resulting in ovarian hyperandrogenism. At the same time, the levels of FSH are abnormally low, causing granulosa cells to not develop properly. Low levels of FSH reduce the activity of aromatase (CYP19A1), resulting in decreased conversion of androgens to estradiol and a buildup of androgen-rich small antral follicles [14]. These follicles are unable to reach the dominant stage, causing multifollicular morphology in the ovaries. Hyperandrogenemia itself also causes further increased GnRH pulsatility in a positive feedback loop that maintains neuroendocrine dysfunction and chronic anovulation (Figure 1).

### 3.2. Insulin Resistance, Metabolic Syndrome, and Hyperandrogenism

**Figure 2:**
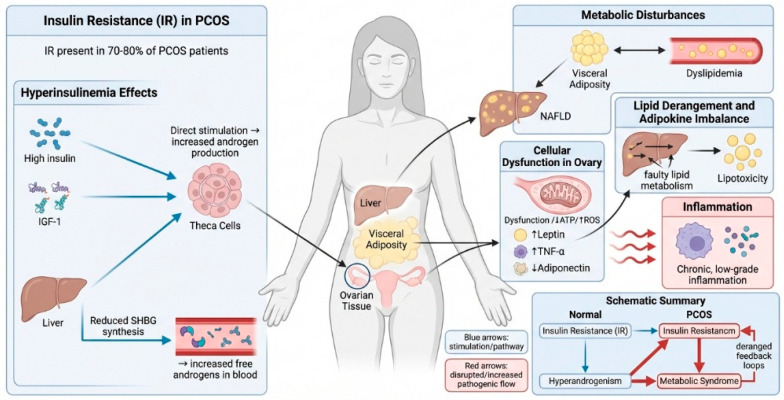
Molecular pathway of insulin resistance, metabolic syndrome, and hyperandrogenism in PCOS. Insulin resistance and compensatory hyperinsulinemia contribute to ovarian hyperandrogenism and metabolic syndrome in PCOS. Increased insulin and IGF-1 signaling augment androgen production in theca cells and inhibit hepatic SHBG synthesis, resulting in elevated free circulating androgens. Insulin resistance also promotes visceral obesity, dyslipidemia, metabolic dysfunction-associated steatotic liver disease (MASLD), lipotoxicity, adipokine imbalance, and chronic low-grade inflammation. Mitochondrial malfunction in the ovaries and oxidative stress exacerbate follicular function, creating a self-reinforcing cycle that connects metabolic and reproductive disorders in PCOS.

Insulin resistance (IR) is present in 70–80% of women with PCOS, independent of BMI, and leads to compensatory hyperinsulinemia [15]. Hyperinsulinemia acts directly on ovarian theca cells through insulin and insulin-like growth factor-1 (IGF-1) receptor-mediated signaling to stimulate androgen production [16]. Additionally, hyperinsulinemia suppresses hepatic synthesis of sex hormone-binding globulin (SHBG), leading to increased circulating free androgens and clinical hyperandrogenism [17]. IR also contributes to visceral obesity, which is the key driver of metabolic dysregulation [18]. Increased visceral adiposity contributes to dyslipidemia and promotes metabolic dysfunction-associated steatotic liver disease (MASLD), further contributing to systemic metabolic dysregulation [19]. IR and metabolic stress in ovarian tissue also contribute to cellular dysfunction, with impaired mitochondrial oxidative phosphorylation, reduced ATP production, and increased production of ROS [20]. Mitochondrial dysfunction impairs granulosa cell function and follicular development. Dysregulated lipid metabolism also contributes to lipotoxicity and adipokine imbalance, with increased leptin and pro-inflammatory cytokines such as TNF-α, as well as decreased adiponectin [21]. This endocrine-metabolic disorder promotes chronic low-grade inflammation, perpetuating both insulin resistance and ovarian dysfunction. The schematic diagram summarizes the pathogenic feedback loops in PCOS, in which insulin resistance promotes hyperandrogenism and metabolic syndrome, which in turn reciprocally exacerbate insulin resistance (Figure 2).

### 3.3. Ovarian Microenvironment and Autophagy Impairment

**Figure 3:**
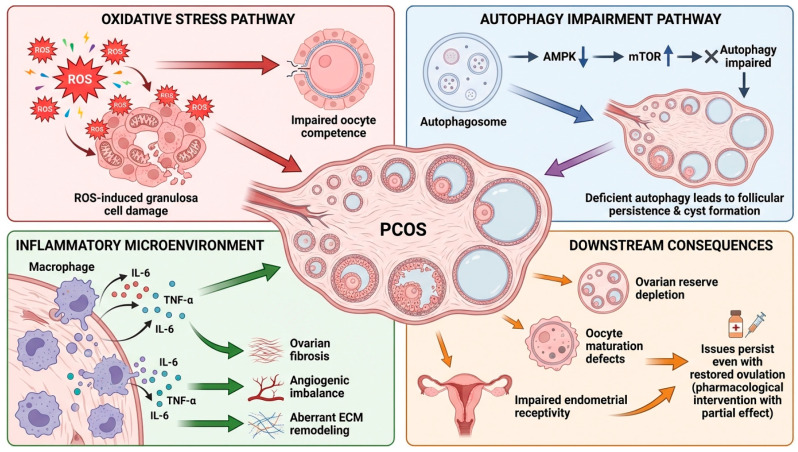
Mechanism of ovarian microenvironment, oxidative stress, and autophagy impairment. Excessive reactive oxygen species formation causes granulosa cell damage and mitochondrial malfunction, resulting in diminished oocyte competence. Dysregulation of the AMPK-mTOR pathway inhibits autophagy, leading to follicular persistence and cyst development. Simultaneously, macrophage infiltration and increased levels of IL-6 and TNF-α create a persistent inflammatory ovarian milieu, fostering fibrosis, angiogenic dysregulation, and abnormal extracellular matrix remodeling. The interrelated pathways result in ovarian reserve depletion, abnormalities in oocyte maturation, and compromised endometrial receptivity, leading to ongoing reproductive failure in PCOS.

The ovarian microenvironment, particularly oxidative stress, autophagy impairment, and inflammatory signaling, plays a pivotal role in PCOS pathogenesis [22]. Dysregulated microenvironmental cues in PCOS ovaries converge, inducing follicular dysfunction and contributing to the multifaceted clinical manifestations of the syndrome (Figure 3). Excessive ROS production in PCOS ovaries promotes a chronic oxidative stress milieu that directly impairs granulosa cell function and mitochondrial integrity [23]. ROS-mediated mitochondrial dysfunction compromises oxidative phosphorylation, leading to diminished ATP production and increased apoptotic signaling within granulosa cells [24]. This mitochondrial distress negatively affects oocyte-somatic cell communication, ultimately reducing oocyte competence and developmental capacity.

Oxidative stress indicators include malondialdehyde (MDA), 8-hydroxy-2′-deoxyguanosine (8-OHdG), total antioxidant capacity (TAC), and superoxide dismutase (SOD). Inflammatory mediators such as IL-6, TNF-α, CRP, and macrophage-associated signaling markers; and autophagy-related metrics including the LC3-II/I ratio, Beclin-1, p62/SQSTM1, and AMPK–mTOR pathway activity [25,26]. Mitochondrial function indicators and accessible biomarkers generated from follicular fluid have been referenced. Significantly, many of these markers have potential in research contexts, but the majority are not yet standardized for regular clinical application and necessitate more validation in longitudinal human studies [27]. The use of biochemical, imaging, and possibly follicular fluid-derived markers may yield a more thorough evaluation of genuine ovarian recovery beyond the mere ovulatory state [28].

In addition, aberrant autophagy signaling is a hallmark of PCOS ovaries. The AMPK-mTOR axis, a critical regulator of autophagy, is dysregulated, with decreased AMPK activation and sustained mTOR signaling [29]. This dysregulation suppresses autophagosome formation and impairs autophagic flux, inhibiting the physiological removal of dysfunctional follicles and cellular debris [30]. As a result, follicles fail to undergo atresia and persist as cystic structures. This failure of normal follicular turnover leads to an accumulation of small antral follicles, a characteristic feature of PCOS ovarian morphology [31]. Simultaneously, the ovarian stroma becomes a chronic inflammatory microenvironment, characterized by macrophage infiltration and increased secretion of proinflammatory cytokines such as interleukin-6 (IL-6) and tumor necrosis factor-α (TNF-α) [32]. This chronic inflammation contributes to ovarian fibrosis, angiogenic imbalances, and aberrant extracellular matrix remodeling, further compromising follicular architecture and ovarian vascularization. The combined structural and biochemical changes progressively lead to ovarian dysfunction. Together, oxidative stress, defective autophagy, and chronic inflammation converge, leading to downstream pathological consequences in PCOS [33]. These sequelae include ovarian reserve depletion, oocyte maturation defects, and endometrial receptivity impairment, ultimately contributing to subfertility and adverse reproductive outcomes. Importantly, these aberrations may persist even when ovulation is restored pharmacologically. This finding underscores the notion that simply restoring ovulation does not fully correct ovarian dysfunction in PCOS.

Recent research indicates that disruption of the AMPK-mTOR signaling pathway may not be consistently observed in all phenotypes of PCOS [34]. In obesity-related and insulin-resistant PCOS, chronic food surplus, hyperinsulinemia, and inflammatory signaling are expected to enhance sustained mTOR activation and diminish AMPK activity, thereby hindering autophagic flux [35]. Conversely, thin PCOS patients may demonstrate less severe metabolic changes, and while alterations in oxidative stress and autophagy have been noted, the degree of AMPK-mTOR imbalance seems to be less significant [36]. Due to the scarcity of phenotype-stratified human data, additional comparison investigations are necessary to ascertain if AMPK-mTOR dysregulation is a universal signature or a mechanism specific to certain subtypes in PCOS [36].

### 3.4. Role of Oxidative Stress in the Pathophysiology of PCOS

Oxidative stress is widely acknowledged as a critical pathogenic element in PCOS, connecting metabolic dysfunction to compromised ovarian physiology. Women with PCOS demonstrate increased levels of ROS and diminished antioxidant capacity in both systemic circulation and follicular fluid [37]. Excessive generation of ROS may arise from mitochondrial malfunction, metabolic stress driven by hyperglycemia, lipotoxicity, and persistent low-grade inflammation [38]. These mechanisms collectively disturb redox homeostasis and establish a pro-oxidative ovarian microenvironment. Oxidative stress directly impacts the functionality of granulosa and theca cells at the cellular level. Mitochondrial fragmentation and compromised oxidative phosphorylation diminish ATP availability, undermining follicular growth and steroidogenic equilibrium [39]. ROS-induced damage to mitochondrial DNA and cellular membranes activates apoptotic signaling pathways, adversely affecting oocyte-somatic cell communication and diminishing oocyte competence [40]. Oxidative stress exacerbates insulin resistance by activating stress-sensitive signaling pathways, including NF-κB and JNK, hence intensifying hyperinsulinemia-induced androgen excess [41]. Moreover, oxidative stress engages with aberrant autophagy in PCOS. Modifications in AMPK-mTOR signaling may hinder the normal elimination of damaged mitochondria, resulting in the buildup of dysfunctional organelles and increased ROS production [42]. This feed-forward cycle leads to follicular arrest, stromal fibrosis, and chronic inflammation in the ovary. These data collectively indicate that oxidative stress serves as a fundamental mechanistic link among endocrine dysregulation, metabolic abnormalities, and reproductive failure in PCOS, emphasizing redox modulation as a possible, albeit still unexplored, treatment approach.

### 3.5. Clinical and Phenotypic Signs of PCOS

**Figure 4:**
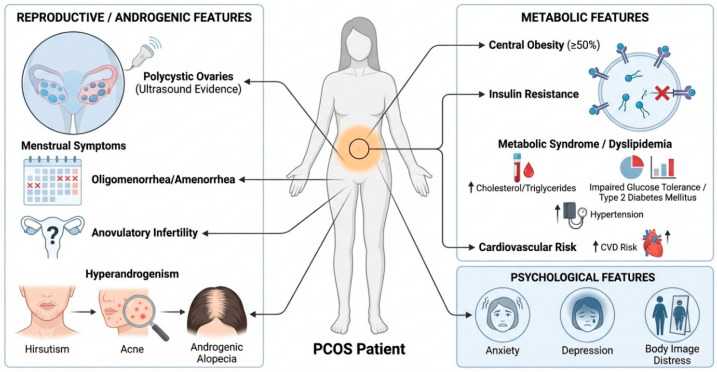
Clinical and phenotypic signs of PCOS. The primary clinical and phenotypic aspects of PCOS include reproductive, androgenic, metabolic, and psychosocial characteristics. Reproductive and androgenic indicators encompass polycystic ovarian morphology identified via ultrasonography, menstrual irregularities including oligomenorrhea or amenorrhea, and anovulatory infertility. Hyperandrogenism clinically presents as hirsutism, acne, and androgenic alopecia. Metabolic characteristics are significant and encompass insulin resistance, central obesity in nearly half of patients, dyslipidemia marked by elevated cholesterol and triglyceride levels, impaired glucose tolerance or type 2 diabetes mellitus, and hypertension, all of which collectively heighten the long-term risk of cardiovascular disease. Alongside physical and metabolic irregularities, psychological consequences such as anxiety, sadness, and body image-related unhappiness are commonly noted, significantly impacting quality of life. The many clinical and phenotypic characteristics collectively illustrate the systemic nature of PCOS, underscoring the necessity for thorough diagnostic and treatment strategies.

The most common clinical signs and symptoms of PCOS are oligomenorrhea or amenorrhea, anovulatory infertility, and ultrasound evidence of polycystic ovaries, along with clinical hyperandrogenism, such as hirsutism, acne, and androgenic alopecia (Figure 4). Insulin resistance is often observed, with up to 50% of patients with PCOS having central obesity, which is often resistant to traditional weight loss regimens [43]. Dyslipidemia, impaired glucose tolerance, type 2 diabetes mellitus, and hypertension have also been recognized as metabolic features of PCOS and place patients at risk for increased long-term cardiovascular disease [44]. Anxiety, depression, and body image distress are common psychological problems in PCOS that are largely underrecognized.

## 4. Modern Treatment Strategies for Polycystic Ovary Syndrome

A modern approach to PCOS care has evolved from treating separate symptoms to providing mechanism-centered as well as individualized interventions with fertility considerations in mind. As a heterogeneous endocrine-metabolic disease, evolving treatment guidelines now advocate for phenotype-driven, stratified treatment based on reproductive intentions, metabolic risk, and future health.

### 4.1. Personalized Lifestyle and Metabolic Optimization

Lifestyle and metabolic optimization are now established as the first-line approach to managing PCOS, across all phenotypes, regardless of BMI and fertility aspirations [45]. Tailored to individual metabolic derangements, current interventions are evidence-based and directly target key features of PCOS: insulin resistance, neuroendocrine imbalance, and low-grade inflammation. Nutritional interventions are paramount, with low-glycemic index diets and Mediterranean diets being preferred, due to their beneficial effects on insulin signaling, inflammation, and androgen levels [46]. These diets are also known to increase ovulation frequency and improve lipid profiles. The focus in dietary recommendations has shifted from mere calorie restriction to improving metabolic health through better nutrient timing, glycemic control, and dietary composition.

Exercise is a key intervention for metabolic remodeling in PCOS, with a combination of resistance training and aerobic activity shown to improve insulin sensitivity, reduce visceral fat, and enhance glucose uptake in skeletal muscle [47]. Resistance training is highlighted for its benefits on basal metabolic rate and androgen metabolism, while aerobic activity supports cardiovascular health and inflammation. The exercise regimens are personalized based on the metabolic phenotype, fitness level, and ovulatory response. Achieving optimal body weight is crucial, with a 5–10% weight loss in overweight and obese women being associated with the restoration of ovulation and improvements in hyperandrogenism and fertility [48]. In lean women with PCOS, the focus is on insulin sensitivity and hormonal regulation, rather than weight loss.

The role of sleep and stress in PCOS management is gaining recognition. Poor sleep, irregular circadian rhythm, and high psychological stress can contribute to increased cortisol levels, exacerbate insulin resistance, and disrupt the HPO axis [49]. Behavioral modifications, including maintaining consistent sleep patterns and employing stress-reduction techniques, are recommended. Digital health interventions, such as AI-based coaching and wearable device integration, are being increasingly adopted [50]. These tools offer personalized insights by combining data from menstrual tracking, glucose monitoring, activity levels, and lifestyle factors, enabling real-time, data-driven recommendations for lifestyle modifications. In summary, lifestyle and metabolic optimization in PCOS care involves personalized, evidence-based interventions targeting diet, exercise, body composition, and lifestyle factors. These measures directly address the underlying pathophysiology of PCOS and are crucial for restoring hormonal balance, improving reproductive health, and reducing long-term health risks.

Digital health technologies, especially AI-driven coaching platforms, are progressively being investigated as adjunctive resources for the long-term management of PCOS [51]. Considering that PCOS is a persistent illness necessitating ongoing lifestyle changes, digital treatments provide a scalable and systematic approach to improve adherence and tailor care. AI-enabled applications can incorporate menstrual tracking, nutritional records, physical activity data, continuous glucose monitoring, and metrics collected from wearables, including heart rate variability and sleep patterns [52]. These systems can produce personalized suggestions for meal timing, exercise intensity, stress management, and ovulation prediction by analyzing multidimensional data in real time. From a feasibility standpoint, women with PCOS may especially benefit due to the syndrome’s metabolic variability and shifting hormone levels [53]. Digital platforms enable regular monitoring without necessitating several clinic visits, thus enhancing accessibility and continuity of service. Nevertheless, pragmatic factors continue to hold significance. Digital literacy, the expense of wearable devices, data security, patient participation, and sustained adherence constitute possible obstacles [54]. Moreover, existing evidence predominantly stems from pilot studies and behavioral intervention programs, while randomized controlled trials specifically assessing AI-driven coaching in PCOS are scarce [55]. AI-driven digital therapies seem viable and encouraging as supplementary resources in the therapy of PCOS; nevertheless, additional validation in varied demographics and practical clinical environments is necessary to determine cost-effectiveness and long-term effects [56].

### 4.2. Pharmacological Management Based on Clinical Goals

#### 4.2.1. Management of Hyperandrogenism and Menstrual Irregularity

For women with PCOS who are not seeking to become pregnant in the short term, contemporary pharmacologic management is goal-directed, typically involving treatments for cycle regulation, endometrial protection, and amelioration of hyperandrogenic symptoms and hirsutism [57]. Combined oral contraceptive pills (COCPs) are the preferred first-line therapy, as they mitigate several drivers of PCOS with a single treatment. Benefits include suppression of pituitary gonadotropin secretion (which inhibits LH-driven androgen synthesis in theca cells), increased hepatic sex hormone-binding globulin (SHBG; which lowers free testosterone), and predictable withdrawal bleeding that obviates the risk of endometrial hyperplasia [58]. Contemporary guidelines no longer recommend a single “best” formulation, but most favor lower doses of ethinyl estradiol and careful consideration of cardiovascular risk.

Combined oral contraceptives (COCs) used for women with PCOS often comprise ethinyl estradiol or estradiol derivatives in conjunction with a synthetic progestin [59]. The estrogen component inhibits hypothalamic-pituitary gonadotropin secretion, thereby diminishing luteinizing hormone-induced ovarian androgen synthesis and elevating hepatic sex hormone-binding globulin, which reduces circulating free testosterone levels [60]. Contemporary formulations predominantly utilize reduced estrogen dosages to mitigate thromboembolic and metabolic hazards [61]. The metabolic and androgenic characteristics of combined oral contraceptives are significantly determined by the specific progestin utilized [59]. Third- and fourth-generation progestins, including desogestrel, norgestimate, and drospirenone, demonstrate reduced androgenic or anti-androgenic activity and are frequently favored for PCOS patients exhibiting hyperandrogenic symptoms [62]. Drospirenone possesses anti-mineralocorticoid and anti-androgenic characteristics that may be advantageous for acne and hirsutism [63]. Conversely, higher androgenic progestins may negatively impact lipid metabolism or insulin sensitivity in predisposed individuals [64]. Consequently, the selection of contraceptives in PCOS must be tailored according to the metabolic risk profile and reproductive objectives.

Systemic anti-androgens may be added to COCPs if hirsutism or acne persists after an adequate trial. Spironolactone (androgen receptor antagonism plus inhibition of androgen action at the pilosebaceous unit) is the most common option, but finasteride (5α-reductase inhibitor that reduces dihydrotestosterone formation) is another choice for hirsutism [65]. Because anti-androgens can cause fetal undervirilization, guidelines recommend avoiding anti-androgen monotherapy unless effective contraception is also used. Importantly, these therapies do not fully reverse insulin resistance or long-term cardiometabolic risk, so they should be used in conjunction with lifestyle and metabolic optimization. Principal pharmacological interventions for hyperandrogenism and menstrual irregularities to treat PCOS are presented in Table 1.

#### 4.2.2. Metabolic and Insulin-Targeted Therapy

Metabolic and insulin-targeted therapy is a fundamental component of contemporary PCOS management, as insulin resistance is a primary contributor to hyperandrogenism, anovulation, and prolonged cardiometabolic risk [72]. In lean PCOS, modest abnormalities in insulin signaling may enhance ovarian androgen production and impede follicular development, rendering metabolic correction clinically significant across phenotypes [73]. Modern methodologies emphasize treatments that enhance insulin sensitivity, diminish compensatory hyperinsulinemia, and indirectly restore endocrine function.

Metformin is the predominant insulin-sensitizing medication utilized in PCOS, especially among individuals with overweight, obesity, prediabetes, or a risk of type 2 diabetes [74]. Metformin diminishes hepatic gluconeogenesis and enhances peripheral glucose uptake, resulting in lowered circulating insulin levels [75]. This reduction subsequently inhibits insulin-mediated androgen production in theca cells and may elevate hepatic sex hormone-binding globulin (SHBG), thereby reducing free testosterone levels. Metformin can enhance menstrual regularity, metabolic parameters, and ovulatory function in specific patients, and it is frequently utilized in conjunction with lifestyle modifications or ovulation induction therapy in fertility-oriented treatment [76].

Concurrently, inositol isomers, particularly myo-inositol (MI) and D-chiro-inositol (DCI), are gaining preference owing to their physiological function as insulin second messengers [77]. MI is closely associated with ovarian function and oocyte quality, whereas DCI affects glycogen production and metabolic signaling [78]. Supplementation with MI, either alone or in meticulously adjusted MI:DCI ratios, has been linked to higher insulin sensitivity, decreased androgen levels, increased ovulatory frequency, and improved reproductive results, with overall acceptable tolerability [79].

In the context of obesity-related PCOS, recent pharmacological alternatives encompass GLP-1 receptor agonists, which facilitate weight reduction by diminishing hunger and enhancing glycemic regulation [80]. GLP-1-based therapy may diminish visceral adiposity and enhance insulin sensitivity, thereby alleviating hyperandrogenism and promoting menstrual regularity [81], especially in those with pronounced metabolic syndrome. Metabolic and insulin-targeted therapy facilitates a transition from mere symptom alleviation to endocrine-metabolic balance, tackling the underlying mechanisms that perpetuate PCOS and enhancing both reproductive and long-term health results (Table 2).

### 4.3. Fertility-Focused Modern Treatments in PCOS

In women with PCOS who are actively trying to conceive, care in the modern era is focused on restoring predictable ovulation and improving oocyte quality while minimizing treatment-associated risk, especially risk of ovarian hyperstimulation syndrome (OHSS) [86]. Contemporary international guidelines recommend letrozole as the first-line pharmacologic treatment for ovulation induction in anovulatory infertility due to PCOS when no other causes of infertility are present. Letrozole is an aromatase inhibitor which may increase endogenous FSH stimulation, leading to monofollicular development with subsequent improved ovulation and live-birth rates compared with older strategies in many clinical settings [87]. When oral ovulation induction fails or is contraindicated, gonadotropins are given with a low-dose individualized protocol as appropriate for baseline ovarian reserve and monitored by serial ultrasound to minimize the risk of multifollicular development and OHSS. For those patients who go on to pursue assisted reproductive technologies (ART), contemporary practice often includes mild stimulation strategies, GnRH antagonist protocols, and evidence-based OHSS prevention strategies, such as individualized dosing and trigger strategies for patients at high-risk of OHSS. Advanced approaches in IVF are increasingly incorporating artificial intelligence-supported tools. These include AI-augmented prediction of ovulation based on longitudinal clinical and wearable data, computerized ovarian stimulation modeling to guide dosing, and deep-learning methods to assess embryos from time-lapse imaging to help improve embryo selection and pregnancy prediction. Fertility-focused modern treatment for PCOS is shifting from an “ovulation induction only” strategy toward a precision, safety-first approach for reproductive optimization. Contemporary treatments for PCOS focus on fertility, mechanisms, and primary actions. Results are presented in Table 3.

### 4.4. Novel and Next-Generation Approaches

In the era of next-generation treatments for PCOS, the management of the disease is changing quickly. New therapies target the underlying pathophysiology rather than treating the symptoms of PCOS. Strategies under investigation or in development include ovarian-directed therapy, restoration of the ovarian microenvironment, and artificial intelligence (AI)-based precision medicine, all of which may lead to improved endocrine function, reproductive outcomes, and long-term metabolic health.

#### 4.4.1. Intelligent Drug Delivery

To improve translational clarity, it is essential to acknowledge that innovative medication delivery techniques in PCOS are predominantly in the preclinical or first exploratory phase. Nanoparticle-based systems have shown better pharmacokinetics and targeted tissue accumulation in reproductive and metabolic models, but there is not much direct evidence from well-defined patient cohorts with PCOS [90]. Key translational obstacles encompass variability in ovarian targeting efficiency, inadequate long-term safety evidence, potential off-target accumulation, industrial scalability, and regulatory approval processes [91]. Moreover, the diversity of PCOS traits may affect therapy outcomes. Consequently, stringent in vivo validation, toxicity assessment, and regulated clinical trials are essential prior to clinical application. Emerging technologies such as nanomedicine offer promising approaches for targeted drug delivery and improved therapeutic efficiency in PCOS. Nanoparticle-based systems may enhance bioavailability and ovarian targeting; however, current evidence remains largely preclinical, and further validation through well-designed clinical studies is required before routine clinical application.

Critical limitations to the current pharmacotherapeutic paradigm for PCOS include poor drug bioavailability, off-target drug toxicity, and variable ovarian exposure. Nanoparticle and nanofiber platforms show promise in improving ovarian drug targeting and enabling controlled, sustained release [92]. Nanocarriers like liposomes, polymeric nanoparticles (e.g., PLGA, PEGylated systems), solid lipid nanoparticles, dendrimers, and exosomes can encapsulate therapeutic payloads such as insulin sensitizers, antioxidants, anti-inflammatory agents, and anti-androgens to prevent degradation and improve cellular uptake [93,94]. Surface modification with specific ligands (e.g., follicle-stimulating hormone receptor ligands or hyaluronic acid) could further enhance selective localization in granulosa or theca cell compartments, increasing therapeutic efficacy and reducing off-target effects.

Nanofiber-based systems provide additional benefits by serving as localized depots with programmable release kinetics. Electrospun nanofiber scaffolds can be engineered to deliver low-dose therapeutics in a time-controlled manner, potentially supporting endometrial receptivity or peri-ovarian microenvironment conditioning [95]. Controlled release platforms also enable combination therapy, such as the co-delivery of metformin with antioxidants or anti-androgens, to simultaneously modulate insulin resistance, oxidative stress, and androgen excess [96]. In summary, intelligent delivery systems represent a forward-thinking approach to “precision pharmacology” in PCOS, with the potential to achieve sustained ovarian restoration with reduced side effects.

Recent advances in nanomedicine highlight the potential of ovarian-targeted delivery platforms to overcome pharmacokinetic limitations observed with conventional PCOS therapies [90]. It has been demonstrated that nanoparticle-based systems can markedly enhance cellular absorption, extend circulation duration, and facilitate tissue-specific accumulation via surface ligand modification [97]. These platforms employ polymers like PLGA and PEGylated carriers to improve stability, diminish immune clearance, and facilitate regulated drug release kinetics [98]. Crucially, the use of receptor-targeting compounds improves selective localization within ovarian tissue, namely in granulosa and theca cell compartments, thus optimizing therapeutic efficacy and reducing systemic exposure [99]. Alongside passive targeting, systems responsive to stimuli such as pH, oxidative stress, or inflammatory microenvironments are being developed [100]. Such systems are especially pertinent in PCOS, where heightened oxidative stress and modified metabolic signaling establish a unique ovarian environment. Controlled-release methods may provide synchronized medication delivery aligned with distinct phases of the follicular cycle, hence enhancing follicular dynamics and oocyte competency [101]. These improvements indicate that intelligent drug administration is a viable translational technique capable of facilitating disease-modifying therapy instead of solely symptomatic relief in the management of PCOS.

#### Intelligent Drug Delivery in PCOS: Current Evidence and Translational Constraints

Nanotechnology-based drug delivery systems have emerged as a promising strategy to enhance therapeutic precision; however, their application in PCOS remains largely at a preclinical stage with limited disease-specific validation. Recent studies using PCOS animal models, particularly letrozole- and dehydroepiandrosterone (DHEA)-induced models, have demonstrated that nanoparticle-mediated delivery of bioactive compounds such as curcumin, resveratrol, and quercetin can improve insulin sensitivity, reduce oxidative stress, and partially restore ovarian morphology [102,103,104]. For example, curcumin-loaded nanoparticles have shown enhanced bioavailability and improved metabolic and inflammatory profiles compared to free compounds in PCOS rodent models [105,106]. Similarly, resveratrol nanoformulations have been reported to modulate steroidogenesis and reduce androgen levels [107].

Despite these encouraging findings, no nanoparticle system has yet been clinically validated specifically for PCOS treatment, and ovarian-targeted delivery remains a major challenge. Currently, most nanocarriers rely on passive distribution rather than PCOS-specific targeting ligands, and there is a lack of validated receptors or biomarkers uniquely overexpressed in PCOS ovaries that can be exploited for selective delivery [108]. This significantly limits target efficiency and raises concerns regarding off-target accumulation and endocrine disruption.

Furthermore, much of the rationale for nanomedicine in PCOS is extrapolated from oncology or metabolic disease models, where targeted delivery systems are more advanced. While such cross-disciplinary insights are valuable, their direct translation to PCOS requires caution due to the unique endocrine and reproductive microenvironment of the ovary.

Key translational barriers include limited long-term safety data, variability in ovarian biodistribution, scalability of nanocarrier production, and regulatory approval challenges. Therefore, although intelligent drug delivery systems hold potential for improving therapeutic specificity, their clinical application in PCOS will require robust in vivo validation, identification of ovary-specific targets, and well-designed clinical trials.

#### 4.4.2. Targeting Ovarian Microenvironment Dysfunction

A significant conceptual advancement in PCOS treatment is the acknowledgment that ovarian dysfunction is influenced not only by circulating hormones but also by local microenvironmental disease. Ovaries affected by PCOS frequently have increased reactive oxygen species, mitochondrial fragmentation, compromised oxidative phosphorylation, persistent stromal inflammation, fibrosis, and dysfunctional autophagy [22]. These alterations injure granulosa cells, hinder oocyte-somatic cell communication, diminish oocyte competence, and undermine endometrial receptivity, leading to enduring subfertility despite pharmacological induction of ovulation [109].

Emerging therapeutics increasingly focus on restoring the ovarian microenvironment via antioxidants and mitochondrial-targeted strategies. Compounds include coenzyme Q10, melatonin, N-acetylcysteine, resveratrol, and other redox-modulating agents that are under investigation to mitigate oxidative stress, maintain mitochondrial membranes, and enhance follicular bioenergetics [110]. In addition to antioxidant therapy, focusing on the autophagy mechanism has become a promising mechanistic method. PCOS is linked to impaired AMPK-mTOR signaling, which hinders autophagic flux and the regulation of follicular atresia, thereby facilitating follicular persistence and cystogenesis [111]. Modulators that augment AMPK activation or modulate mTOR activity may restore autophagy, facilitate follicle turnover, diminish stromal fibrosis, and increase metabolic balance in the ovary [112]. This method redefines treatment as ovarian tissue reprogramming instead of hormonal masking, aligning PCOS therapy with wider advances in regenerative and metabolic medicine.

#### 4.4.3. Artificial Intelligence-Driven Precision Medicine

Artificial intelligence is revolutionizing PCOS management through phenotype-based treatment selection, predictive modeling, and real-time therapy optimization. Due to the many endotypes of PCOS, including hyperandrogenic, insulin-resistant, inflammatory, lean, or ovarian aging-driven, artificial intelligence provides a means to stratify patients more precisely than conventional criteria alone [56]. Machine learning systems can assimilate multidimensional data, encompassing hormonal panels (AMH, LH/FSH, testosterone, insulin), metabolic markers, ovarian ultrasound patterns, inflammatory indices, microbiome signals, and longitudinal clinical history to discern actionable subtypes and forecast responses to specific interventions [113].

Ovulation forecasting, metabolic risk prediction, diagnostic categorization, and imaging-based phenotyping have been the main areas of recent research utilizing artificial intelligence in PCOS [56]. To improve diagnostic discrimination between PCOS and non-PCOS cohorts, machine learning models-such as logistic regression classifiers, random forests, gradient boosting algorithms, and support vector machines-have been trained on hormonal parameters like insulin levels, LH, FSH, testosterone, AMH, metabolic biomarkers, and anthropometric data [114]. Numerous research claims that in internally validated datasets, classification accuracies and area under the curve (AUC) values surpass 0.80 to 0.90. Deep learning techniques have also been applied to ultrasound image processing to reduce operator dependence and interobserver variability in automated follicle counting and polycystic ovarian morphology detection [115]. Artificial intelligence (AI)-assisted systems for ovulation prediction and embryo selection that integrate wearable data and longitudinal hormonal data have shown encouraging predictive power in reproductive care settings [116]. Most of the research is still retrospective, single-center, and relies on small sample sizes, though. Standardized reporting, model interpretability, external validation, and prospective clinical testing are still lacking. Furthermore, only a few AI systems have made it from proof-of-concept to regular clinical use. Therefore, before AI-driven decision support in PCOS is widely used in clinical treatment, further extensive, multicenter validation studies are necessary, even though preliminary data suggests that it is feasible.

AI facilitates personalized fertility management, encompassing ovulation forecasting, enhanced stimulation protocols in IVF, and embryo selection through computer vision and time-lapse imaging [117]. Digital health platforms and wearable devices offer continuous physiological data, including heart rate variability, sleep metrics, stress indicators, and glucose trends, which AI may convert into meaningful lifestyle recommendations and chronotherapy-based dosage regimens [118]. In the foreseeable future, AI may facilitate tailored nanomedicine by refining nanoparticle design parameters and releasing kinetics according to a patient’s endocrine-metabolic condition, hence expediting the implementation of intelligent delivery systems in clinical PCOS management [119].

In addition to ovulation tracking and embryo selection, artificial intelligence is progressively included in comprehensive precision medicine frameworks pertinent to PCOS [56]. Machine learning algorithms have been utilized on endocrine and metabolic information to discern previously unidentified PCOS endotypes, facilitating categorization according to insulin resistance severity, androgen excess patterns, inflammatory profiles, or ovarian reserve metrics [114]. Predictive models are being created to assess individual reactions to pharmacological treatments like letrozole, metformin, or GLP-1 receptor agonists, thereby minimizing empirical treatment choices and expediting therapeutic optimization [120]. AI-assisted ultrasound analysis is an emerging application in which deep learning models improve the identification and quantification of polycystic ovarian morphology, follicle count, and stromal volume with less operator reliance [121]. Furthermore, multi-omics integration platforms that amalgamate genomes, transcriptomics, metabolomics, and microbiome markers are initiating the discovery of molecular subtypes of PCOS that could inform targeted therapy approaches [122]. Digital coaching systems based on reinforcement learning can dynamically modify food, physical activity, and sleep interventions, utilizing continuous data generated from wearables [123]. These tools provide adaptive treatment models that progress alongside hormonal swings and metabolic alterations, advancing PCOS management towards predictive, preventative, and entirely individualized medicine.

#### Artificial Intelligence in PCOS: Evidence-Based Summary and Clinical Translation

Artificial intelligence (AI) applications in PCOS have expanded rapidly, particularly in diagnosis, phenotyping, metabolic risk prediction, and reproductive outcome optimization. Several machine learning (ML) models, including support vector machines (SVMs), random forests (RFs), k-nearest neighbors (KNNs), and logistic regression, have been developed using clinical, hormonal, and metabolic datasets [124]. For example, studies using publicly available PCOS datasets (e.g., UCI Machine Learning Repository) and hospital-based cohorts have reported diagnostic accuracies ranging from 85% to 95%, with area under the curve (AUC) values often exceeding 0.85 [125,126]. These models typically incorporate features such as LH, FSH, testosterone, insulin levels, BMI, and menstrual irregularity patterns.

Deep learning approaches have also been applied to ultrasound imaging for automated detection of polycystic ovarian morphology. Convolutional neural networks (CNNs) have demonstrated improved follicle detection accuracy and reduced interobserver variability compared to conventional manual assessment [127]. In reproductive medicine, AI-assisted embryo selection and ovulation prediction models integrating time-lapse imaging and hormonal data have shown promising predictive performance, with AUC values reported between 0.80 and 0.93 in assisted reproductive technology (ART) settings [128].

Despite these advances, most studies are based on retrospective, single-center datasets with limited sample sizes and lack external validation. Few models have undergone prospective clinical testing or regulatory evaluation. Additionally, heterogeneity in diagnostic criteria and dataset composition limits generalizability across diverse PCOS phenotypes [129].

To improve clarity and reproducibility, a summary table of representative AI studies in PCOS, including model type, dataset, performance metrics, and validation status, has been incorporated (Table 4). While current findings support the feasibility of AI-driven decision support in PCOS, large-scale multicenter studies and standardized validation frameworks are essential before routine clinical implementation.

### 4.5. Dietary Interventions for Metabolic and Endocrine Modulation in PCOS

Dietary modification is a crucial aspect of contemporary PCOS care, especially as insulin resistance is a primary cause of hyperandrogenism and ovulatory dysfunction [136]. Nutritional solutions are increasingly customized to metabolic phenotype rather than exclusively emphasizing calorie restriction [137]. Low-glycemic index (GI) and Mediterranean-style diets are among the most substantiated methods, as they promote insulin sensitivity, diminish systemic inflammation, improve lipid profiles, and increase ovulatory frequency [138]. These dietary patterns prioritize whole grains, lean proteins, unsaturated fats, vegetables, and foods high in antioxidants, which together promote hormonal and metabolic equilibrium.

**Figure 5:**
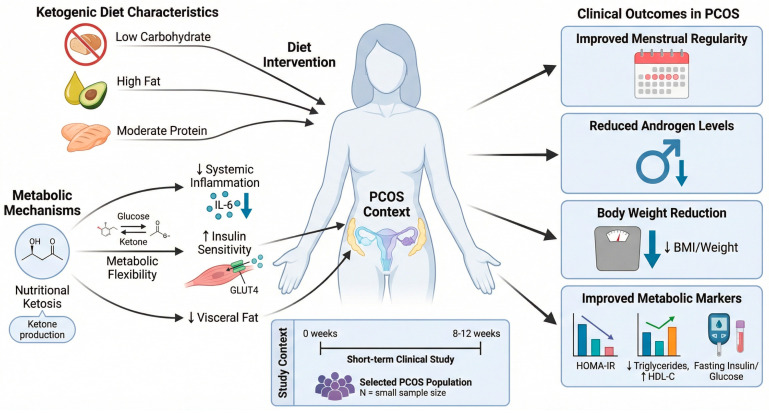
Effect of ketogenic diets on PCOS mechanisms and clinical outcomes. Nutritional ketosis and ketone body synthesis are promoted by a ketogenic diet with low carbohydrate, high fat, and moderate protein. Carbohydrate restriction lowers glucose and insulin levels, improves GLUT4-mediated glucose absorption, promotes metabolic flexibility, and reduces visceral adiposity. In PCOS, metabolic adjustments reduce systemic inflammation, particularly IL-6 levels, and improve insulin signaling. In selected PCOS populations, short-term studies of 8–12 weeks have shown improvements in menstrual regularity, circulating androgen levels, weight, body mass index, and metabolic parameters like HOMA-IR, triglycerides, HDL-C, and fasting insulin and glucose. Evidence from modest, short-term trials suggests potential benefits for endocrine and metabolic regulation, emphasizing the need for bigger, longer-term studies.

Ketogenic diets, defined by significant carbohydrate limitation and elevated fat consumption, induce nutritional ketosis and metabolic flexibility, potentially improving insulin sensitivity, decreasing systemic inflammation, and aiding in the elimination of visceral fat [139]. Recent clinical trials indicate that short-term ketogenic therapies may enhance menstrual regularity, androgen levels, body weight, and metabolic indicators in some groups with PCOS [140]. Schematic representation of Figure 5 the metabolic mechanisms and clinical effects of ketogenic dietary intervention in PCOS.

Recent research corroborates the efficacy of low-carbohydrate and ketogenic diets in some populations of PCOS, especially among women with obesity and significant insulin resistance [141]. By markedly diminishing carbohydrate consumption, ketogenic methods decrease circulating insulin levels, enhance lipolysis, and may diminish ovarian androgen production by lowering the stimulation of theca cells [142]. Short-term investigations have indicated enhancements in weight, insulin resistance indicators, menstrual regularity, and serum androgen concentrations [143]. Nonetheless, long-term safety, sustainability, and reproductive effects necessitate additional research. Consequently, dietary therapies for PCOS must be tailored to the individual’s metabolic profile, reproductive objectives, patient preferences, and ability to comply, accompanied by vigilant physician oversight when employing restrictive methods.

## 5. Evidence Grading Framework and Strength of Recommendations

To improve methodological rigor and transparency, the strength of evidence discussed in this narrative review was evaluated using a structured framework adapted from established hierarchies in evidence-based medicine, including principles from the GRADE (Grading of Recommendations Assessment, Development and Evaluation) approach and conventional levels of clinical evidence. Given the scope of this review, which synthesizes multidisciplinary and emerging data, the grading system was applied as a qualitative interpretative tool rather than a formal systematic evaluation. The application of the evidence grading framework to major therapeutic strategies is summarized in Table 5, where each intervention is explicitly categorized (Grade A-D) based on the strength and type of supporting evidence.

To improve methodological openness and elucidate the robustness of supporting data, a systematic evidence-grading approach for the principal therapeutic options is examined. To employ evidence grading based on ideas derived from established hierarchies in evidence-based medicine, including international clinical guideline approaches. Interventions were classified into four tiers. Grade A was designated to strategies endorsed by high-quality international clinical practice guidelines, numerous randomized controlled trials (RCTs), or meta-analyses exhibiting consistent efficacy [144]. Examples encompass lifestyle modification as the primary intervention and the use of letrozole for ovulation induction in anovulatory PCOS [145]. Grade B indicates evidence derived from one or more rigorously designed randomized controlled trials (RCTs) or extensive prospective cohort studies, exemplified by metformin for metabolic risk mitigation and specific insulin-targeted interventions [146]. Grade C indicates minimal or varied clinical data, comprising small clinical trials or short-term interventional research, as observed with ketogenic diets and specific nutraceutical strategies [147]. Grade D signifies primarily preclinical, mechanistic, or proof-of-concept data without substantial human validation, exemplified by numerous nanoparticle-based drug delivery technologies and sophisticated AI predictive models [148]. Every grade assignment is explicitly connected to the referenced literature within the manuscript. This paradigm enables readers to differentiate between therapy approved by guidelines and emergent translational discoveries. Next-generation techniques are examined with careful interpretation to prevent exaggerating clinical preparedness. The incorporation of evidence grading enhances the scientific rigor of this review while maintaining its integrative and progressive outlook.

Importantly, each grading assignment was linked to cited literature and interpreted within the context of study quality and translational relevance. To explicitly acknowledge that, as a narrative review, this framework does not include formal risk-of-bias assessment, meta-analytic weighting, or systematic literature selection. Therefore, while the grading provides a structured overview of evidence strength, it should not be interpreted as a definitive ranking of clinical efficacy. This approach aims to balance clarity with caution, recognizing the heterogeneity of PCOS research and the evolving nature of next-generation therapeutic strategies.

## 6. Current Limitations and Future Directions to Treat PCOS

Although advancements have been made in comprehending PCOS, present management is constrained as most treatments focus on alleviating symptoms rather than improving the condition itself. Oral contraceptives modulate bleeding and alleviate androgenic symptoms; however, they may obscure disease activity and fail to rectify insulin resistance, ovarian oxidative stress, or microenvironmental inflammation. Metformin enhances metabolic markers; nevertheless, responses differ, gastrointestinal intolerance diminishes adherence, and reproductive advantages are inconsistent when administered alone [149]. Letrozole-induced ovulation is beneficial for numerous people; nonetheless, it does not rectify underlying follicular dysfunction or endometrial receptivity deficiencies in every instance [87]. In cases of refractory infertility, gonadotropins and IVF enhance the likelihood of pregnancy but introduce additional costs, invasiveness, and the danger of ovarian hyperstimulation syndrome, particularly in high-responding PCOS phenotypes. A significant systemic constraint is the absence of validated biomarkers to ascertain which individuals will respond to certain interventions, leading to trial-and-error prescribing and postponed optimization of reproductive and metabolic outcomes. The variability of PCOS complicates management, as various endotypes-hyperandrogenic, insulin-resistant, inflammatory, lean, or related to ovarian aging-frequently get analogous treatment protocols.

Future directions increasingly emphasize precision phenotyping and mechanism-based repair. Multi-omics profiling, sophisticated imaging, and digital biomarker integration are anticipated to provide enhanced categorization and tailored treatment selection. Therapies aimed at the ovarian microenvironment, such as mitochondrial rescue, oxidative stress mitigation, and autophagy normalization through AMPK-mTOR modulation, could enhance oocyte competency and reproductive lifetime beyond mere ovulation induction [150]. Advanced drug delivery methods, such as nanoparticles and nanofibers, may improve ovarian targeting, facilitate controlled release, and enable combination therapy while minimizing systemic toxicity. Artificial intelligence is expected to enhance decision-making by forecasting treatment responses, optimizing ovulation timing, refining IVF stimulation to mitigate OHSS risk, and facilitating adaptive lifestyle recommendations based on wearable data. The forthcoming generation of PCOS therapy seeks to transition from symptom management to the reversal of endocrine-metabolic dysfunction, the preservation of fertility, and the prevention of long-term cardiometabolic consequences via integrated, personalized care.

Despite increasing interest in improved therapy methods for PCOS, the existing database is still small and varied. Numerous proposed interventions, including nanomedicine and AI-driven methodologies, are predominantly substantiated by preclinical research or retrospective assessments, with scant prospective clinical validation. The inconsistency in diagnostic criteria, limited sample numbers, and absence of standardized outcome measures further restrict generalizability. Furthermore, mechanistic insights are frequently assumed rather than directly evidenced in human cohorts. Although these developing methods exhibit potential, their clinical usefulness remains ambiguous. Comprehensive, extensive, and longitudinal studies are necessary to confirm safety, efficacy, and reproducibility prior to standard clinical application.

## 7. Translational Pathways and Clinical Implementation Challenges in PCOS

Despite significant advances in next-generation therapeutic strategies for PCOS, successful clinical translation requires a structured and phased approach addressing safety, efficacy, and regulatory considerations. For nanoparticle-based drug delivery systems, preclinical studies must be complemented by rigorous toxicological evaluation, including reproductive toxicity, endocrine safety, and long-term biodistribution profiling. Pharmacokinetic and pharmacodynamic characterization, particularly ovarian targeting efficiency and off-target accumulation, are critical prior to Investigational New Drug (IND) approval [151]. Additionally, scalable manufacturing under Good Manufacturing Practice (GMP) conditions and regulatory compliance remain essential barriers [152].

For AI-driven precision medicine, translation from retrospective datasets into clinical practice requires robust external validation, prospective multicenter trials, and demonstration of clinical utility. AI models must address bias, reproducibility, and generalizability across diverse populations. Regulatory frameworks, such as the U.S. Food and Drug Administration (FDA) pathway for Software as a Medical Device (SaMD), emphasize transparency, interpretability, and continuous performance monitoring [153].

Microenvironment-targeted therapies, including modulation of oxidative stress, inflammation, and autophagy, require validated biomarkers for patient stratification and therapeutic monitoring [154]. Long-term safety, especially for pathway-modulating agents, remains insufficiently characterized. Furthermore, combination therapies targeting metabolic and reproductive pathways may face complex regulatory approval processes.

Overall, bridging the gap between experimental innovation and clinical implementation in PCOS necessitates coordinated efforts in translational research, standardized validation frameworks, and regulatory alignment to ensure safety and efficacy in human populations.

## 8. Conclusions

Polycystic ovary syndrome is a complex disorder that results from interactions of endocrine, metabolic, and ovarian microenvironmental derangements. While existing treatments continue to play a significant role, many are symptomatic and do not address the underlying pathogenesis. Next-generation therapies, such as intelligent drug delivery, metabolic reprogramming, and AI-based precision medicine, show promise in restoring ovarian function, improving fertility outcomes, and mitigating long-term metabolic risk. By integrating molecular insights with technological advances, future management of PCOS may evolve from a reactive to a proactive, personalized, and disease-modifying approach.

## Figures and Tables

**Table 1:** Key pharmacologic agents used for hyperandrogenism and menstrual irregularity in PCOS.

Pharmacologic Agents	Molecular Mechanism	Main Clinical Action in PCOS	Expected Effects	Precautions	Ref.
Combined oral contraceptive pills (COCPs)	Suppress GnRH–LH/FSH signaling, ↓ LH-driven theca androgen output; ↑ hepatic SHBG → ↓ free testosterone	First-line for irregular cycles and hyperandrogenism	Enhanced cycle regularity, reduced acne and hirsutism over months, and endometrial protection	Choose based on cardiometabolic and thrombotic risk; no ideal formulation.	[66]
Cyclic progestin (e.g., medroxyprogesterone, micronized progesterone)	Secretory transformation and withdrawal bleeding result from progestin exposure.	Endometrial protection when COCPs are not used	Protects against unopposed estrogen; may ease bleeding.	Not hirsutism-specific; used when estrogen is contraindicated.	[67]
Spironolactone	Androgen receptor antagonism lowers hair follicle and sebaceous gland androgen.	Add-on for hirsutism/acne after COCPs	Lower Ferriman-Gallwey score, improved acne	Need dependable contraception; monitor potassium in some cases.	[68,69]
Finasteride	Inhibits 5α-reductase → ↓ dihydrotestosterone (DHT)	Alternative add-on for hirsutism	Lower hirsutism and hair growth measurements	Teratogenic risk to the male fetus; contraception required	[69]
Topical eflornithine (face)	Inhibits ornithine decarboxylase in hair follicle → slows hair growth	Adjunct for facial hirsutism	Better cosmetic control, slower facial hair development	Combines best with hair removal and/or systemic therapy.	[70]
Metformin (when metabolic risk is present)	Improves insulin signaling; ↓ hepatic gluconeogenesis; indirect androgen lowering via ↓ insulin and ↑ SHBG	Not primary for hirsutism, used for metabolic indications	Glycemic measurements may improve cycles in some	Best for obese/metabolic risk people; not anti-androgen.	[66,71]

Footnotes: COCP (combined oral contraceptive pill), FSH (follicle-stimulating hormone), DHT (dihydrotestosterone), SHBG (sex hormone-binding globulin).

**Table 2:** Metabolic and insulin-targeted therapy to endocrine-metabolic balance, addressing PCOS’s root causes and improving reproductive and long-term health.

Pharmacological Agents	Molecular Mechanism	Therapeutic Action in PCOS	Typical Clinical Effects	Evidence Level in PCOS	Ref
**Metformin (biguanide)**	Increases AMPK signaling, decreases hepatic gluconeogenesis, increases peripheral insulin sensitivity, lowers circulating insulin, indirectly reduces theca androgen synthesis, and may boost SHBG	First-line insulin-sensitizer for metabolic indications, adjuvant for cycle irregularity when COCPs are ineffective, and an adjunct for infertility in certain patients	Reduces metabolic risk, improves insulin resistance, fasting glucose, menstrual cyclicity, and ovulation.	Strong guideline-supported metabolic therapy	[67]
**Myo-inositol (MI)**	Insulin second messenger precursor promotes insulin receptor signaling, ovarian function, and oocyte metabolic competence.	Supporting insulin resistance, ovulation, and fertility	Some trials show improved insulin sensitivity, cycle regularity, and ovulation, and good tolerability.	Mixed evidence, commonly used guidelines acknowledge variable certainty based on outcomes.	[82,83]
**D-chiro-inositol (DCI)**	Glycogen production and metabolic pathways supported by an insulin signaling mediator may minimize hyperinsulinemia-driven androgen excess.	Metabolic support, sometimes combined with MI	May enhance insulin resistance and androgen markers in some cohorts, dose and phenotype dependent.	Mixed evidence, dose, and MI:DCI ratio affect outcomes.	[82]
**MI + DCI combination (physiologic ratio approaches)**	Supporting dual insulin signaling, MI promotes ovarian function and oocyte quality, DCI metabolic signaling, and possible synergy when balanced.	Insulin resistance, ovulatory support, and fertility supplements	Some trials showed improvements in endocrine markers and insulin resistance; variability among studies.	Mixed to moderate evidence; study design affects conclusions.	[82]
**GLP-1 receptor agonists (e.g., liraglutide, semaglutide class)**	GLP-1R activation reduces hunger, energy intake, glycemic management, weight loss, visceral adiposity, insulin sensitivity, and may indirectly lower androgens.	Treatment of obesity-related PCOS and metabolic syndrome frequently involves lifestyle changes and metformin	In obesity-associated PCOS, weight loss, insulin resistance, and cardiometabolic indicators may enhance androgenicity and menstrual regularity.	Growing usage of obesity-associated PCOS necessitates pregnancy planning measures.	[84,85]

Footnotes: AMPK (adenosine monophosphate-activated protein kinase), SHBG (sex hormone-binding globulin), MI (myo-inositol), DCI (D-chiro-inositol), GLP-1R (glucagon-like peptide-1 receptor).

**Table 3:** Therapies for PCOS targeting fertility: mechanisms, principal actions, and outcomes.

Approach	Molecular or Physiologic Mechanism	Clinical Action in PCOS Infertility	Key Outcomes or Advantages	Main Risks or Limits	Ref
Letrozole (first-line ovulation induction)	Aromatase inhibition → ↓ estrogen feedback → ↑ FSH drive and follicular recruitment	Induces ovulation in anovulatory PCOS	Recommended first-line, enhances ovulation and fertility in eligible people	Needs monitoring and timing, not suitable if other infertility factors prevail	[66]
Gonadotropins (individualized low-dose protocols)	Exogenous FSH stimulation of folliculogenesis	After oral induction fails, stimulation controls for timed intercourse or IUI.	Properly dosed ovulation and pregnancy induction	High OHSS and multiple gestation risk without monitoring	[88]
ART with mild stimulation, OHSS prevention strategies	Protocol-controlled ovarian stimulation to limit overreaction	PCOS IVF with safer stimulation, generally antagonist-based	Maintains reproductive potential and reduces OHSS risk	Cost, invasiveness, and still need close monitoring	[88]
AI-supported IVF, ovulation prediction, and embryo selection	Machine and deep learning on clinical data and time-lapse imaging	Helps dosage, embryo ranking, pregnancy prediction	Results prediction and selection consistency may increase	Evidence quality varies; external evaluation and integration are difficult	[89]

Footnotes: FSH (follicle-stimulating hormone), IUI (intrauterine insemination), IVF (in vitro fertilization), OHSS (ovarian hyperstimulation syndrome).

**Table 4:** Summary of artificial intelligence applications in PCOS: models, performance, and validation status.

Study Objective	Dataset/Sample Size	Input Features	AI Model	Key Findings (Performance)	Validation Status	Ref.
PCOS diagnosis prediction	Public dataset (UCI), n ≈ 541	Hormonal (LH, FSH), BMI, insulin, menstrual history	SVM, Random Forest	Accuracy ~90–94%, AUC > 0.88	Internal validation	[130]
Classification of PCOS vs. non-PCOS	Clinical dataset, n ≈ 500	Metabolic + hormonal parameters	Random Forest, KNN	Accuracy ~92%, improved feature selection performance	Internal validation	[130]
PCOS prediction using ensemble learning	Public + clinical dataset, n ≈ 500	Clinical + biochemical	Ensemble ML models	AUC up to 0.95, high sensitivity	Internal validation	[131]
Ultrasound-based PCOS detection	Ultrasound images, n ≈ 200–300	Ovarian imaging features	CNN (Deep Learning)	Improved follicle detection, reduced observer bias	Internal validation	[132]
Ovulation prediction	Clinical longitudinal dataset, n ≈ 200	Hormonal + cycle data	Machine learning model	AUC ~0.85 for ovulation prediction	Internal validation	[133]
Embryo selection in ART	IVF dataset, n > 1000 embryos	Time-lapse embryo imaging	Deep learning (CNN)	AUC 0.80–0.93 for implantation prediction	External validation (limited)	[134]
Metabolic risk prediction in PCOS	Clinical cohort, n ≈ 300	Insulin, glucose, lipid profile	Logistic regression, ML models	Improved prediction of insulin resistance	Internal validation	[135]
PCOS classification and feature ranking	Public dataset, n ≈ 541	Clinical + hormonal	Gradient boosting, RF	Accuracy ~93%, robust feature importance	Internal validation	[114]

**Table 5:** Evidence Grading of Therapeutic Strategies in PCOS Management.

Therapeutic Category	Intervention	Mechanism of Action	Clinical Application	Evidence Level	Key Supporting Evidence
**Lifestyle** **Intervention**	Diet (Low-GI, Mediterranean)	Improves insulin sensitivity, reduces inflammation	First-line management across all PCOS phenotypes	**Grade A**	International guidelines, meta-analyses
	Exercise (aerobic + resistance)	Enhances glucose uptake, reduces visceral fat	Metabolic and reproductive improvement	**Grade A**	RCTs, systematic reviews
**Pharmacological Therapy**	Letrozole	Aromatase inhibition, ↑ FSH	First-line ovulation induction	**Grade A**	Clinical guidelines, RCTs
	Combined Oral Contraceptives (COCs)	Suppress LH, ↑ SHBG	Cycle regulation, ↓ hyperandrogenism	**Grade A**	Clinical guidelines
	Metformin	Activates AMPK, ↓ hepatic glucose output	Insulin resistance, metabolic management	**Grade B**	RCTs, cohort studies
	Inositols (MI/DCI)	Insulin signaling modulation	Ovulatory support, metabolic balance	**Grade B–C**	Mixed RCT evidence
	GLP-1 receptor agonists	Weight reduction, ↑ insulin sensitivity	Obesity-associated PCOS	**Grade B**	Emerging clinical trials
**Fertility** **Treatments**	Gonadotropins	Direct ovarian stimulation	Second-line ovulation induction	**Grade A–B**	Clinical trials
	ART (IVF with mild stimulation)	Controlled follicular recruitment	Infertility management	**Grade A**	Established clinical practice
**Dietary** **Strategies**	Ketogenic diet	↓ insulin, ↑ fat metabolism	Metabolic improvement in select PCOS	**Grade C**	Small clinical trials
**Microenvironment Targeting**	Antioxidants (CoQ10, NAC, resveratrol)	↓ ROS, improves mitochondrial function	Adjunct therapy	**Grade C**	Small trials, mechanistic studies
	AMPK-mTOR modulators	Restores autophagy	Experimental ovarian restoration	**Grade D**	Preclinical evidence
**Nanomedicine**	Nanoparticle drug delivery	Targeted delivery, controlled release	Experimental therapy	**Grade D**	Preclinical models
**Artificial** **Intelligence**	Diagnostic ML models	Pattern recognition (hormonal, metabolic)	PCOS classification	**Grade C–D**	Retrospective studies
	AI in IVF/ovulation prediction	Predictive modeling	Fertility optimization	**Grade C–D**	Limited validation studies

## Data Availability

No new data were created or analyzed in this study. Data sharing is not applicable.
